# Antimicrobial Cathelicidin Peptide LL-37 Inhibits the LPS/ATP-Induced Pyroptosis of Macrophages by Dual Mechanism

**DOI:** 10.1371/journal.pone.0085765

**Published:** 2014-01-16

**Authors:** Zhongshuang Hu, Taisuke Murakami, Kaori Suzuki, Hiroshi Tamura, Kyoko Kuwahara-Arai, Toshiaki Iba, Isao Nagaoka

**Affiliations:** 1 Department of Host Defense and Biochemical Research, Juntendo University Graduate School of Medicine, Tokyo, Japan; 2 Department of Bacteriology, Juntendo University Graduate School of Medicine, Tokyo, Japan; 3 Department of Emergency and Disaster Medicine, Juntendo University Graduate School of Medicine, Tokyo, Japan; University of California Merced, United States of America

## Abstract

Pyroptosis is a caspase-1 dependent cell death, associated with proinflammatory cytokine production, and is considered to play a crucial role in sepsis. Pyroptosis is induced by the two distinct stimuli, microbial PAMPs (pathogen associated molecular patterns) and endogenous DAMPs (damage associated molecular patterns). Importantly, cathelicidin-related AMPs (antimicrobial peptides) have a role in innate immune defense. Notably, human cathelicidin LL-37 exhibits the protective effect on the septic animal models. Thus, in this study, to elucidate the mechanism for the protective action of LL-37 on sepsis, we utilized LPS (lipopolysaccharide) and ATP (adenosine triphosphate) as a PAMP and a DAMP, respectively, and examined the effect of LL-37 on the LPS/ATP-induced pyroptosis of macrophage-like J774 cells. The data indicated that the stimulation of J774 cells with LPS and ATP induces the features of pyroptosis, including the expression of IL-1β mRNA and protein, activation of caspase-1, inflammasome formation and cell death. Moreover, LL-37 inhibits the LPS/ATP-induced IL-1β expression, caspase-1 activation, inflammasome formation, as well as cell death. Notably, LL-37 suppressed the LPS binding to target cells and ATP-induced/P2X_7_-mediated caspase-1 activation. Together these observations suggest that LL-37 potently inhibits the LPS/ATP-induced pyroptosis by both neutralizing the action of LPS and inhibiting the response of P2X_7_ to ATP. Thus, the present finding may provide a novel insight into the modulation of sepsis utilizing LL-37 with a dual action on the LPS binding and P2X_7_ activation.

## Introduction

Sepsis is a systemic response that results from a harmful or damaging host response to infection, and is the most common cause of death in the noncoronary intensive care unit (ICU) [1]. Hospital sepsis mortality has declined in recent years, with advances in care. However, novel and effective therapeutic approach is expected to be developed for treatment of sepsis [2], [3], [4]. In sepsis, the dysregulation of inflammatory/immune responses is responsible for the multiple organ failure, for which over expression of proinflammatory cytokines is a major mechanism. In recent years, much attention has been focused on the mechanism of host cell death, which develops during sepsis and contributes to the dysregulated inflammatory/immune responses [5], [6], [7], [8], [9].

Pyroptosis is a recently identified caspase-1 dependent cell death of macrophages and dendritic cells found in bacterial infection [10]. During pyroptosis, the cells rapidly produce and extracellularly release proinflammatory cytokines (IL-1β and IL-18) [10], [11]. IL-1β is a prototypical proinflammatory cytokine, which stimulates both local and systemic inflammatory/immune responses [12], and acts synergistically with other cytokines to cause tissue injury in sepsis [13]. The processing and release of IL-1β is mediated mainly by caspase-1, which also induces cell death [11]. Of importance, caspase-1 knockout exhibits the protective effect on a murine sepsis model, where the plasma IL-1β level was completely depressed, suggesting a crucial role of caspase-1 in sepsis [14], [15], [16], [17]. Induction of pyroptosis requires the two distinct stimuli, microbial PAMPs (pathogen associated molecular patterns) (such as nucleic acid, lipoproteins and lipopolysaccharide LPS) and endogenous DAMPs (damage associated molecular patterns) (such as uric acid and ATP) [10], [11]. TLRs (Toll like receptors) initiate a signaling cascade that leads to cellular activation (including the upregulation of proinflammatory cytokines), in response to PAMPs. In contrast, in responses to DAMPs, NLRs (Nod like receptors)/NLRPs (Nod like receptor proteins) are recruited for the formation of inflammasome, in which the procaspase-1 is converted to the active caspase-1. Finally, the activated caspase-1 processes and releases IL-1β, and induces cell death in an unidentified mechanism [15], [18], [19].

LPS (lipopolysaccharide) is a major component of the outer membrane of Gram-negative bacteria and acts as a potent inducer of proinflammatory responses in monocytes and macrophages; thus, LPS is recognized as a key molecule in the pathogenesis of Gram-negative sepsis [20]. LPS activates macrophages via the binding to membrane receptors, CD14/TLR4 [20], [21], [22]. Thereafter, the pyroptosis of LPS-primed macrophages is induced by ATP, which is normally concentrated in the living cells but released into the extracellular milieu from dead cells [23]. ATP stimulates a purinergic nucleotide receptor P2X_7_ to trigger the signal to induce the formation of an NLRP3 inflammasome, which is composed of NLRP3, ASC (apoptosis-associated speck-like protein containing a carboxyl-terminal CARD) and caspase-1 [24], [25].

AMPs (antimicrobial peptides) represent the first line of defense against invading pathogens [26], [27]. Cathelicidin family of AMPs have been identified in various mammalian species, and LL-37 is the only known human cathelicidin, cleaved from CAP18 (a cationic antimicrobial polypeptide of 18-kDa) [26], [28]. In addition to its broad spectrum of bactericidal activities, LL-37 has a wide range of inflammatory/immune modulatory actions [29], [30]. We previously revealed that LL-37 neutralizes the action of LPS by directly binding with it [20], [31], [32]. Furthermore, LL-37 and CRAMP (cathelicidin-related AMP, a sole identified murine cathelicidin, an orthologue of human LL-37) have been recently reported to modulate the response of P2X_7_ receptor to ATP [33], [34].

Importantly, LL-37 protects experimental septic animals from death [32], [35], [36]; however the mechanism for the protective action of LL-37 on sepsis remains unclear. Based on the potent actions of LL-37 on LPS and P2X_7_ receptor, we hypothesized that LL-37 may modulate the LPS/ATP-induced pyroptosis of macrophages. The results indicated that LPS/ATP treatment induced the pyroptosis of mouse J774 macrophage-like cells, and LL-37 inhibited the LPS/ATP-induced features of pyroptosis, including IL-1β release, caspase-1 activation and cell death. Moreover, LL-37 repressed the LPS binding to the cells, and the ATP-induced P2X_7_ activation, suggesting that LL-37 suppresses the pyroptosis by both neutralizing the LPS action and inhibiting the P2X_7_ response to ATP. Thus, the present findings indicate a novel aspect of LL-37 for the inhibition of LPS/ATP-induced pyroptosis of macrophages.

## Materials and Methods

### Reagent

LPS (*Escherichia coli* O111:B4), FITC-conjugated-LPS (*Escherichia coli* O111:B4) and ATP were purchased form Sigma-Aldrich (St Louis, MO). Ac-YVAD-CHO, a specific caspase-1 inhibitor was purchased from Peptide Institute (Osaka, Japan). A 37-mer peptide of hCAP18 (LL-37; L^1^LGDFFRKSKEKIGKEFKRIVQRIKDFLRNLVPRTES^37^), was synthesized by the solid-phase method on a peptide synthesizer (model PSSM-8; Shimadzu Scientific Instruments, Kyoto, Japan) by fluorenylmethoxycarbonyl chemistry, as described before [31]. KN-62 and KN-93 were purchased from Calbiochem (Darmstadt, Germany). Anti-mouse CD14 (4C1) and anti-mouse TLR4 (MTS510) monoclonal antibodies were purchased form BD Biosciences (San Jose, California).

### Cell culture

A murine macrophage cell line J774 was purchased from American Type Culture Collection (Manassas, VA) and maintained in DMEM medium (Sigma-Aldrich) supplemented with 10% fetal bovine serum (Sankojunyaku, Tokyo, Japan) and 100 U/ml penicillin/100 µg/ml streptomycin (Nacalai Tesque, Kyoto, Japan) at 37°C in 5% CO_2_. J774 cells (1×10^5^ cells/well) in 48 well-culture plates (Iwaki Brand, Asahi Glass, Tokyo, Japan) were primed with 10 ng/ml LPS for 4 h in the absence or presence of LL-37 (0.01, 0.1 or 1 µg/ml), and then treated with 3 mM ATP for the indicated time periods at 37°C in 5% CO_2_. Thereafter, the supernatants were recovered for the assays of IL-1β and lactate dehydrogenase (LDH).

A human monocytic cell line THP-1 was purchased from American Type Culture Collection and maintained in RPMI-1640 medium (Sigma-Aldrich) supplemented with 10% fetal bovine serum and 100 U/ml penicillin/100 µg/ml streptomycin at 37°C in 5% CO_2_. THP-1 cells (5×10^6^ cells/well) in 10 cm-culture plates were differentiated into macrophages by incubating with 5 ng/ml phorbol 12-myristate 13-acetate (PMA) overnight. Thereafter, the adherent cells were detached and seeded in 48-well-culture plates. Then, the cells were primed with 1 µg/ml LPS for 24 h in the absence or presence of LL-37 (1 µg/ml), and treated with 5 mM ATP for 4 h at 37°C in 5% CO_2_. Thereafter, the supernatants were recovered for the assays of IL-1β and LDH.

### Quantification of IL-1β and LDH

IL-1β 1evels in the supernatants were determined using a commercially available mouse IL-1β ELISA kit (detection limits of 16 pg/ml; R&D Systems, Minneapolis, MN) or human IL-1β ELISA kit (detection limits of 4 pg/ml; R&D Systems), according to the manufacturer's instructions.

LDH activities in the supernatants were determined for the evaluation of cell death; LDH activities in the supernatants and 1% Triton X-100-lysed cells (as a total activity of 100%) were determined using a commercially available LDH assay kit (Takara Bio Inc., Shiga, Japan), according to the manufacturer's instructions.

### Assay for the caspase-1 activation

J774 cells (1×10^6^ cells/well) in 6 well-culture plates were primed with 10 ng/ml LPS for 4 h in the absence or presence of LL-37 (0.01, 0.1 or 1 µg/ml), and then treated with 3 mM ATP for 90 min. Thereafter, the caspase-1 activation was assayed by using a FLICA™ Caspase-1 Assay Kit (Immunochemistry Technologies, Bloomington, MN). Briefly, after the treatment, cells were detached using cell scrapers, washed and suspended in 300 µl DMEM. Then, the cells were labeled with FAM-YVAD-fmk (fluorescent labeled caspase-1 inhibitor that bind with activated caspase-1) by incubation for 1 h at 37°C, washed and finally assayed by flow cytometry, according to the manufacturer's manual using FACSCalibur (BD Biosciences, Rutherford, NJ, USA). In separate experiments, J774 cells were directly treated with ATP in the presence or absence of LL-37 or P2X_7_ antagonist (KN-62 or KN-93), and the caspase-1 activation was evaluated, as described above.

### Assay for the inflammasome formation

The formation of inflammasome was detected by staining with FLICA™ Caspase-1 Assay Kit. J774 cells (5×10^5^ cells/well) were seeded over a 12-mm glass coverslip (Fisher Science, Pittsburg, PA) in 24 well-plates overnight. Cells were primed with 10 ng/ml LPS for 4 h in the absence or presence of LL-37 (1 µg/ml), and then treated with 3 mM ATP for 90 min. Thereafter, the cells were washed with DMEM and stained with FAM-YVAD-fmk and Hoechst 33342. Then, the coverslips were mounted on a slide glass by using an aqueous media Vectashield (Vector Labs, Burlingame, CA), and the cells were photographed with a fluorescence microscope system Axioplan 2 (Carl Zeiss, Jena, Germany). The percentage of inflammasome forming cells with activated caspase-1 was determined by counting at least 200 Hoechst 33342 positive cells.

### Assay for the mRNA expression of IL-1β

J774 cells (1×10^6^ cells/well) in 6 well-culture plates were primed with 10 ng/ml LPS for 4 h in the absence or presence of LL-37 (0.01, 0.1 or 1 µg/ml), and then treated with 3 mM ATP for 90 min. Thereafter, the cells were detached using cell scrapers, and then total-RNA was purified using an RNeasy plus mini kit (Qiagen, CA) and QIAshredder (Qiagen) to remove contaminated DNA, according to the manufacturer's protocol. RT-PCR was performed using a ReverTra-Plus-TM RT-PCR kit (Toyobo, Osaka, Japan) in a thermal cycler (Eppendorf Mastercycler Gradient PCR System, Eppendorf, Hamburg, Germany) for evaluating the mRNA expression of IL-1β and GAPDH. In brief, cDNA was synthesized by reverse transcription of total RNA (1 µg/ml) using ReverTra Ace reverse transcriptase and oligo(dT)20. To discriminate mRNA-derived PCR products from genomic DNA-derived products, the intron-spanning PCR primers were used with the annealing temperature of 60°C and cycle number (Table S1). PCR products were resolved by 2% agarose gel electrophoresis in 1×Tris-borate-EDTA buffer and stained with SYBR Safe DNA Gel Stain (Life Technologies Corporation, Gaithersburg, MD). In preliminary experiments, we tried to semi-quantitatively detect mRNA by using different cycle number of PCR. The results indicated that the amounts of RT-PCR products increased dependently on the cycle number. Thus, we decided to measure the mRNA levels by RT-PCR with the cycle number indicated in Table S1. The detected bands were quantified using Bio Doc-It™ System (UVP, Upland, CA).

### Measurement of LPS binding to target cells

J774 cells (5×10^5^ cells/ml) were incubated with FITC-conjugated LPS (1 µg/ml) in the absence or presence of LL-37 (0.01, 0.1 or 1 µg/ml), 4C1 (10 µg/ml, anti-mouse CD14 antibody) or MTS510 (40 µg/ml, anti-mouse TLR4 antibody) in DMEM containing 10% FBS for 15 min at 37°C. After washing the cells with PBS, the binding of FITC-LPS was analyzed by flow cytometry (FACSCalibur), and the median fluorescence intensity was determined.

### Statistic analyses

Data are shown as mean ± standard error. Statistical test was performed by one-way analysis of variance (ANOVA), followed by Bonferroni's multiple comparison test (GraphPad Prism; GraphPad Software, San Diego, CA). A P value of <0.05 was considered to be significant.

## Results

### ATP treatment of LPS-primed J774 cells induces pyroptosis

**Figure 1:**
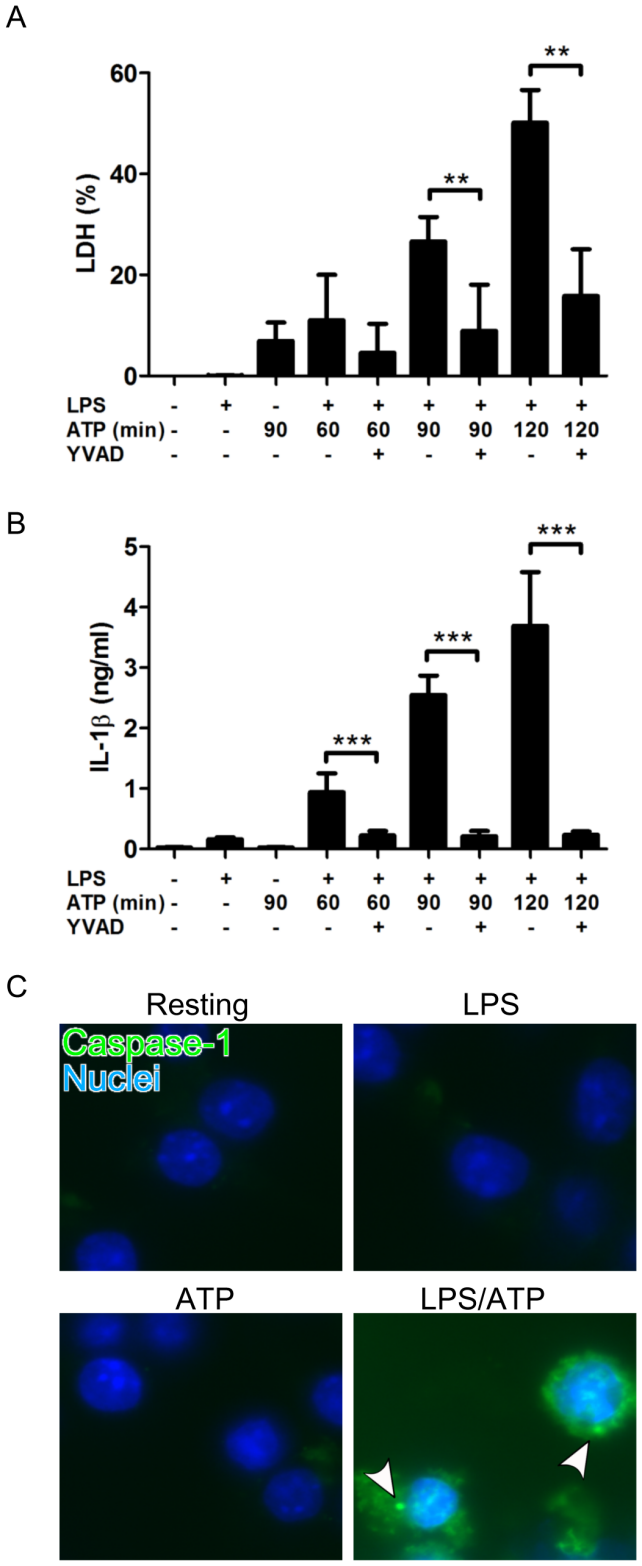
Effect of LPS and ATP treatment on the pyroptosis of J774 cells. Macrophage-like J774 cells were primed with 10 ng/ml LPS for 4 h, and then treated with 3 mM ATP for the indicated time periods in the absence or presence of 20 µM Ac-YVAD-CHO, a caspase-1 specific inhibitor. Cells were also incubated with LPS or ATP alone, or without LPS and ATP (Resting). Thereafter, the supernatants were recovered for the assays of IL-1β (A) and LDH (B). IL-1β 1evels were determined using a commercially available mouse IL-1β ELISA kit. LDH activities in the supernatants and 1% Triton X-100-lysed cells (as a total activity of 100%) were determined using a commercially available LDH assay kit. Data shows the mean ± SD of 3 separate experiments. Values are compared between the absence and presence of Ac-YVAD-CHO. **P<0.01, ***P< 0.001. (C) J774 cells were primed with 10 ng/ml LPS for 4 h and then treated with 3 mM ATP for 90 min. Thereafter, the cells were stained with FAM-YVAD-fmk (a fluorescent labeled caspase-1 inhibitor for inflammasome staining, green) and Hoechst 33342 (for nuclear staining, blue) and photographed with a fluorescence microscope system. Arrowheads indicate the inflammasomes containing the activated caspase-1. Images of cells are representative of 3 separate experiments.

We first examined the effect of LPS and ATP treatment on macrophages by measuring the release of IL-1β and LDH. Mouse macrophage-like J774 cells were primed with 10 ng/ml LPS for 4 h, and then treated with ATP for indicated periods up to 120 min. We confirmed that LPS treatment alone for 4 h did not essentially elicit the release of IL-1β and LDH (Fig. 1A and B). Similarly, ATP treatment alone for 90 min did not induce the IL-1β release; however it slightly but significantly released LDH compared with resting without LPS/ATP treatment (P<0.01) (Fig. 1B). Furthermore, ATP treatment of LPS-med J774 cells significantly induced the release of IL-1β and LDH in a time dependent manner. Interestingly, Ac-YVAD-CHO, a caspase-1 specific inhibitor, inhibited both the LPS/ATP-induced release of IL-1β and LDH, suggesting that the LPS/ATP-induced IL-1β release and cell death (LDH release) are caspase-1 dependent. In addition, we detected the formation of inflammasome by staining the activated caspase-1 with FAM-YVAD-fmk, a fluorescence labeled caspase-1 inhibitor. As shown in Fig. 1C, LPS or ATP treatment alone did not induce the inflammasome formation; however, the ATP treatment of LPS-primed J774 cells considerably increased the inflammasome formation. These observations indicate that the treatment with both LPS and ATP induces the pyroptosis (IL-1β release, cell death accompanied with inflammasome formation) of J774 cells, as reported using mouse bone marrow-derived macrophages and human macrophage-like THP-1 cells [37], [38]. Separately, we confirmed that LPS treatment alone induced the TNF-α production, and the production was not affected by ATP treatment and caspase-1 inhibitor (Fig. S1), suggesting that the TNF-α production is independent of caspase-1 signaling, which is involved in the LPS/ATP-induced pyroptosis.

### LL-37 inhibits the LPS/ATP-induced pyroptosis

Furthermore, we investigated the effect of LL-37 on the LPS/ATP-induced pyroptosis of J774 cells. Thus, J774 cells were primed with 10 ng/ml LPS in the absence or presence of LL-37 (0.01∼1 µg/ml), and treated with 3 mM ATP for 90 min. Then, the effect of LL-37 on the IL-1β release, IL-1β mRNA expression, LDH release, caspase-1 activation and inflammasome formation was evaluated.

**Figure 2:**
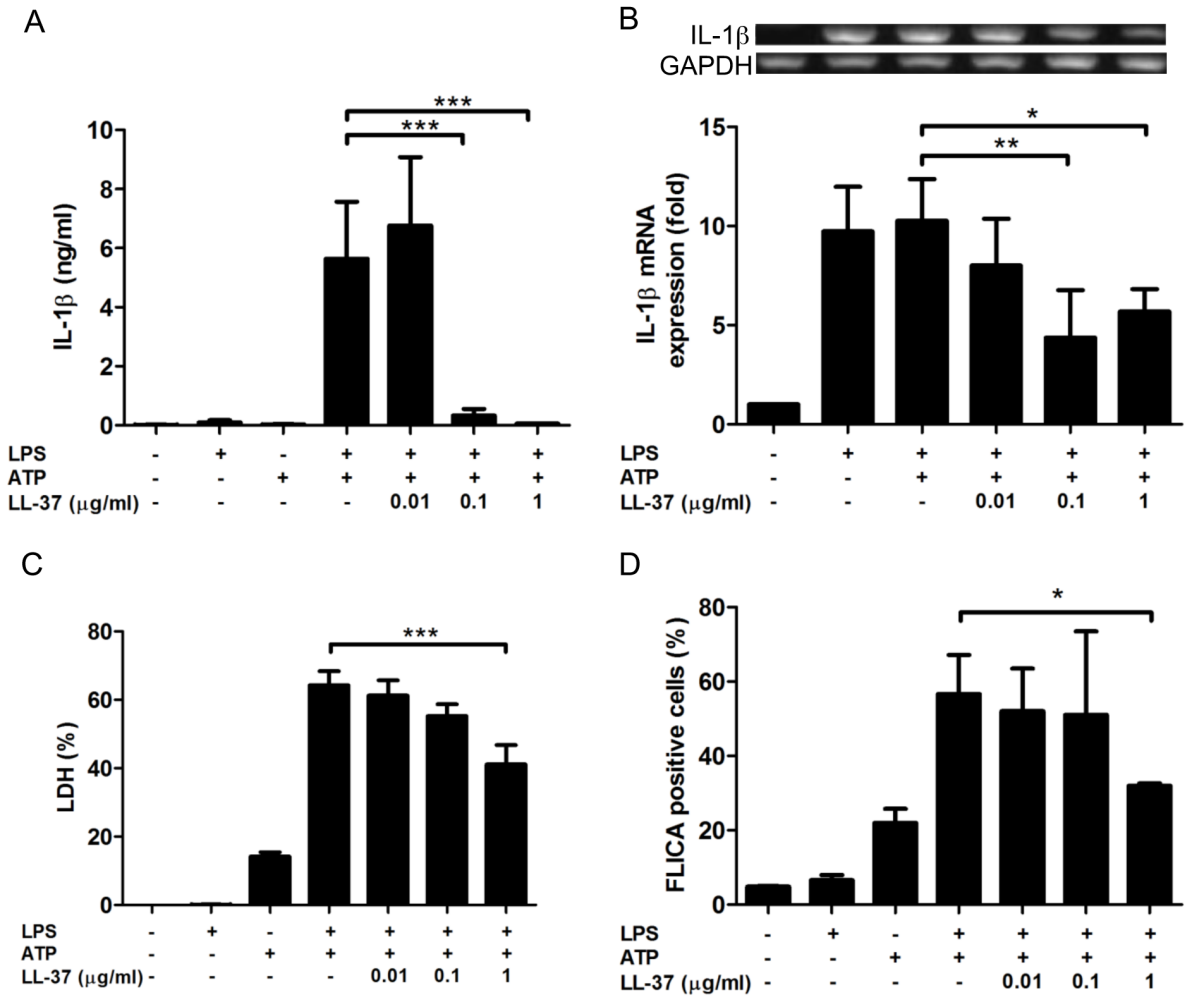
Effect of LL-37 on the LPS/ATP-induced pyroptosis of J774 cells. J774 cells were primed with 10/ml LPS for 4 h, and then treated with 3 mM ATP for 90 min in the absence or presence of LL-37 (0.01, 0.1 or 1 µg/ml). Cells were also incubated with LPS or ATP alone, or without LPS and ATP. Thereafter, the supernatants were recovered for the assays of IL-1β (A) and LDH (C), and the cells were used for the assays of IL-1β mRNA expression (B) and caspase-1 activation (D). IL-1β mRNA expression were determined by RT-PCR and expressed as fold increase relative to resting cells incubated without LPS, ATP and LL-37; the caspase-1 activation was assayed by flow cytometry using FAM-YVAD-fmk (a fluorescent labeled inhibitor of caspase-1, FLICA) that irreversibly binds with activated caspase-1, and expressed as the percentage of FLICA positive cells. Data shows the mean ± SD of 3-5 separate experiments. Values are compared between the absence and presence of LL-37 among LPS/ATP-treated cells. *P<0.05, **P<0.01, ***P<0.001. Images of RT-PCR are representative of 3-5 separate experiments.

Importantly, LL-37 significantly inhibited the LPS/ATP-induced IL-1β production by J774 cells at 0.1 and 1 µg/ml, but not 0.01 µg/ml (Fig. 2A). Next, we examined the effect of LL-37 on the IL-1β mRNA expression. IL-1β mRNA expression was upregulated by LPS alone but not affected by the addition of ATP (Fig. 2B). Notably, LL-37 at 0.1 and 1 µg/ml but not 0.01 µg/ml significantly suppressed the mRNA expression. Furthermore, we investigated the effect of LL-37 on the cell death of LPS/ATP-treated J774 cells, based on the release of LDH. LL-37 suppressed the cell death (LDH release) induced by both LPS and ATP treatment in a dose dependent manner, and significantly inhibited at 1 µg/ml (Fig. 2C). In addition, we examined the effect of LL-37 on the activation of caspase-1 in LPS/ATP-treated J774 cells by flow cytometry. Treatment with ATP but not LPS alone slightly but significantly induced the caspase-1 activation (P<0.001), and the treatment with both LPS and ATP further increased the caspase-1 activation (Fig. 2D). Of importance, LL-37 significantly suppressed the activation of caspase-1 activation at 1 µg/ml (Fig. 2D).

**Figure 3:**
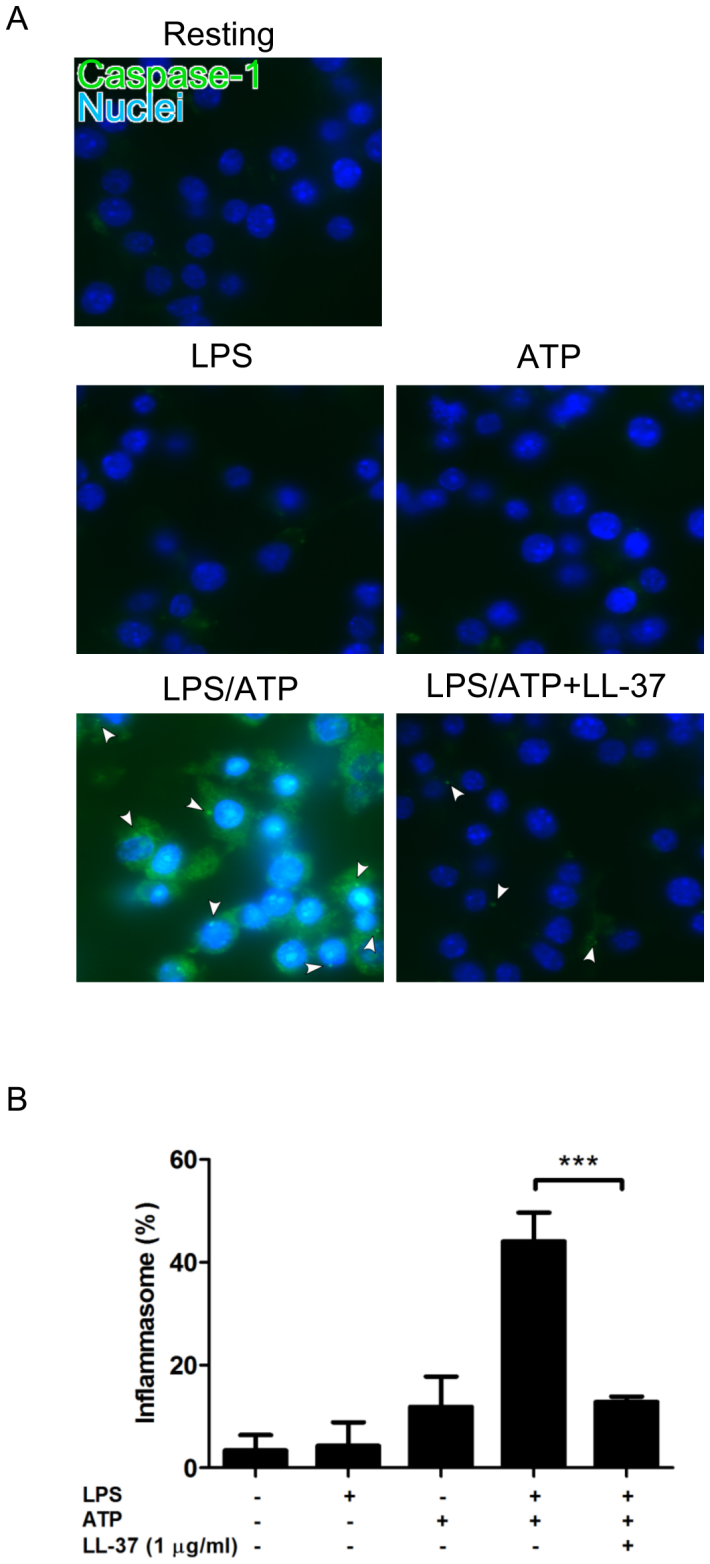
Effect of LL-37 on the LPS/ATP-induced inflammasome formation in J774 cells. J774 cells were primed with 10/ml LPS for 4 h, and then treated with 3 mM ATP for 90 min in the absence or presence of LL-37 (1 µg/ml). Cells were also incubated with LPS or ATP alone, or without LPS and ATP (Resting). Thereafter, the cells were stained with FAM-YVAD-fmk (a fluorescent labeled caspase-1 inhibitor) and Hoechst 33342 (A). Arrowheads indicate the inflammasomes containing the activated caspase-1. Furthermore, the percentage of inflammasome-containing cells with activated caspase-1 was determined by counting at least 200 Hoechst positive cells (B). Data shows the mean ± SD of 3-5 separate experiments. Values are compared in the LPS/ATP-treated cells between the absence and presence of LL-37. ***P<0.001. Images cells are representative of 3–5 separate experiments.

Further, we examined the effect of LL-37 on the formation of inflammasome by using FAM-YVAD-fmk, and the percentage of inflammasome-containing cells with activated caspase-1 was determined. Treatment with ATP but not LPS alone slightly increased the inflammasome formation (P = 0.08), and the treatment with both LPS and ATP further increased the inflammasome formation to 45% among the cells (Fig. 3). Importantly, LL-37 (1 µg/ml) considerably suppressed the inflammasome formation.

These observations indicate that LL-37 potentially inhibits the LPS/ATP-induced pyroptosis (mRNA expression and release of IL-1β, caspase-1 activation and cell death) of J774 cells and inflammasome formation. Interestingly, mRNA expression and release of IL-1β was significantly suppressed by 0.1 µg/ml LL-37; however, cell death, caspase-1 activation and inflammasome formation were not inhibited by 0.1 µg/ml LL-37 but significantly inhibited by 1 µg/ml LL-37, indicating that the suppressive effects of LL-37 are different among the features of LPS/ATP-induced pyroptosis.

Furthermore, we examined the effect of LL-37 on the pyroptosis of human macrophages using THP-1 cells. Thus, PMA-differentiated THP-1 cells were primed with 1 µg/ml LPS in the absence or presence of LL-37 (1 µg/ml), and then treated with 5 mM ATP for 4 h. LPS/ATP treatment similarly induced the release of IL-1β and LDH. Importantly, LL-37 significantly suppressed the LPS/ATP-induced IL-1β release and LDH from human macrophage-like cells (Fig. S2A and B).

### LL-37 inhibits the binding of LPS to J774 cells

**Figure 4:**
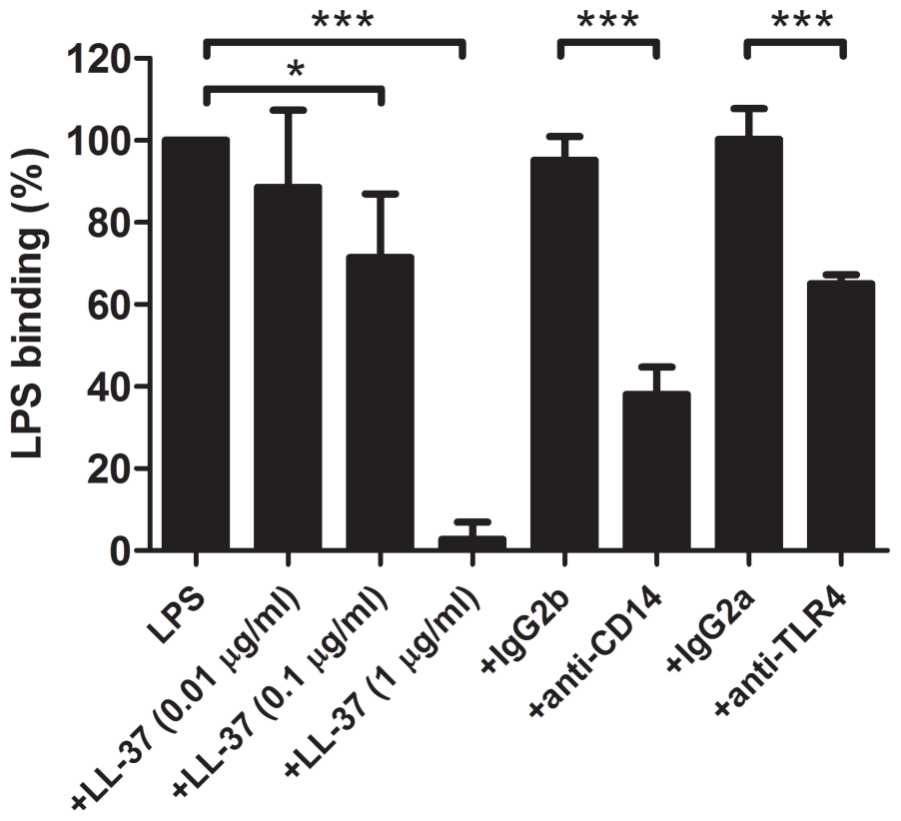
Effect of LL-37 on the LPS binding to J774 cells. J774 cells were suspended in DMEM containing 10% FBS, and incubated with 1 µg/ml FITC-LPS at 37°C for 15 min in the absence or presence of LL-37 (0.01, 0.1 or 1 µg/ml), anti-mouse CD14 monoclonal antibody (4C1, 10 µg/ml), anti-mouse TLR4 monoclonal antibody (MTS510, 40 µg/ml) or isotype control IgG (IgG2b and IgG2a). The binding of LPS was analyzed by flow cytometry, and the median fluorescence intensity was determined. The LPS binding was expressed as the percentage of that with FITC-LPS alone. Data shows the mean ± SD of 3 separate experiments. Values are compared between the absence and presence of LL-37, anti-CD14 monoclonal antibody or anti-TLR4 monoclonal antibody. **P<0.01, ***P<0.001.

To elucidate the mechanism for the suppressive actions of LL-37 on LPS/ATP-induced pyroptosis of J774 cells, we firstly examined the effect of LL-37 on the LPS binding to J774 cell by using FITC-LPS in the absence or presence of LL-37 (0.01-1 µg/ml). As shown in Fig. 4, LL-37 inhibited the LPS binding to J774 cells in a dose dependent manner. LL-37 partially inhibited the binding at 0.1 µg/ml and completely inhibited the binding at 1 µg/ml (P<0.01 at 0.1 µg/ml; P<0.001 at 1 µg/ml). Separately, we revealed that the LPS binding to J774 cells is significantly inhibited by not only anti-CD14 monoclonal antibody but also anti-TLR4 monoclonal antibody, indicating that the LPS binding to J774 cells is mostly mediated by an LPS receptor CD14/TLR4. These observations suggest that LL-37 inhibits the binding of LPS to its receptor CD14/TLR4 to modulate the LPS/ATP-induced pyroptosis.

### LL-37 inhibited the ATP-induced caspase-1 activation

**Figure 5:**
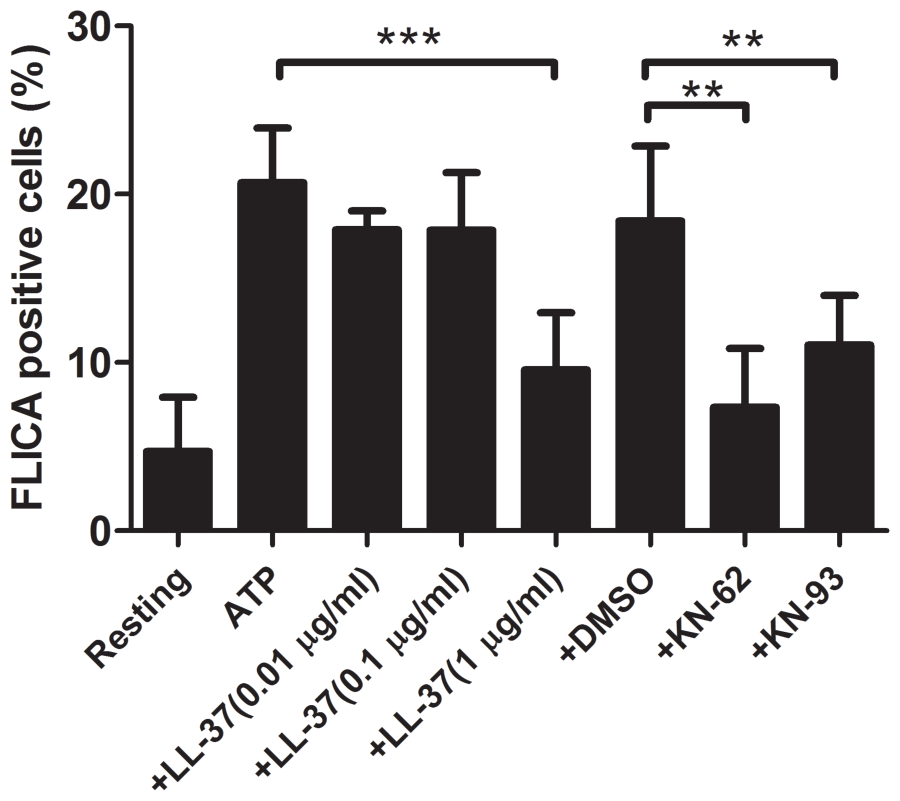
Effect of LL-37 on the ATP-induced caspase-1 activation in J774 cells. J774 cells were treated with 3-37 (0.01, 0.1 or 1 µg/ml), P2X_7_ antagonists (1 µM KN-62 and 1 µM KN-93) or dimethylsulfoxide (DMSO, a solvent for KN-62 and KN-93, 0.1%). Cells were also incubated without ATP, LL-37 and P2X_7_ antagonists (Resting). Thereafter, the caspase-1 activation was assayed by flow cytometry using FAM-YVAD-fmk (FLICA), and expressed as the percentage of FLICA positive cells. Data shows the mean ± SD of 3 separate experiments. Values are compared between the absence and presence of LL-37 or P2X_7_ antagonists among ATP-treated cells. **P<0.01, ***P<0.001.

Secondly, we assessed the effect of LL-37 on the ATP-induced P2X_7_ responses by detecting the caspase-1 activation. As shown in Fig. 5, ATP treatment alone substantially activated the caspase-1 in J774 cells. KN-62 (1 µM) and KN-93 (1 µM), P2X_7_ receptor antagonists, significantly suppressed the ATP-induced caspase-1 activation, suggesting that the ATP activates the caspase-1 via the action on P2X_7_ receptor. Interestingly, LL-37 markedly inhibited the ATP-induced caspase-1 activation at 1 µg/ml, suggesting that LL-37 is able to inhibit the ATP-induced P2X_7_ responses

## Discussion

Pyroptosis is a caspase-1 dependent cell death, associated with proinflammatory cytokine production, and is considered to play a crucial role in the dysregulation of inflammatory/immune responses in sepsis [10], [11], [14], [15]. Pyroptosis is induced by the two distinct stimuli, microbial PAMPs and endogenous DAMPs [10], [11]. In response to PAMPs, TLRs initiate a signaling cascade that leads to the upregulation of proinflammatory cytokines. In contrast, in responses to DAMPs, NLRP inflammasome is formed, where caspase-1 is activated, and finally active caspase-1 processes/releases IL-1β and induces cell death [15], [18], [19].

In this study, we utilized LPS and ATP as a PAMP and a DAMP, respectively, and revealed that the stimulation of macrophage-like J774 cells with LPS and ATP induces the features of pyroptosis including the expression and release of IL-1β, activation of caspase-1, inflammasome formation and cell death. Furthermore, LPS is assumed to be acting on the CD14/TLR4, since the LPS binding to the cells was significantly inhibited by the neutralizing anti-CD14 and anti-TLR4 monoclonal antibodies (Fig. 4). By contrast, ATP is acting on the nucleotide receptor P2X_7_, since the action of ATP, as assessed by the activation of caspase-1, was substantially suppressed by the P2X_7_ antagonist (Fig. 5). Importantly, the stimulation with LPS or ATP alone was enough for the induction of the expression of IL-1β mRNA or activation of caspase-1, respectively, whereas the stimulation with both LPS and ATP was required for the production and release of IL-1β, formation of inflammasome and induction of cell death.

Cathelicidin-related AMPs have a crucial role in innate immune defense [29], [30], [39]. Importantly, it is reported that human cathelicidin LL-37 exhibits the protective effect on the septic animal models [32], [35], [36], and has a potential to modulate the action of LPS and activation of P2X_7_ [20], [33], [40]. Thus, the effect of LL-37 on the LPS/ATP-induced pyroptosis of J774 cells was investigated. The data indicated that LL-37 inhibits the LPS/ATP-induced expression of IL-1β mRNA and protein, activation of caspase-1, as well as cell death (Fig. 2-3). Moreover, LL-37 suppressed the LPS binding to target cells and ATP-induced caspase-1 activation. Together these observations suggest that LL-37 potently inhibits the LPS/ATP-induced pyroptosis by both neutralizing the action of LPS and inhibiting the response of P2X_7_ to ATP.

Previously, we revealed that LL-37 inhibits the LPS-induced TNF-α production by murine macrophage-like RAW264.7 cells and the LPS-induced apoptosis of human endothelial cells through the neutralization of LPS actions [20], [31]. LL-37 can directly bind with LPS and inhibit the interaction of LPS with LPS-binding protein (LBP), thereby preventing the transfer of LPS to its receptor CD14 [20], [41]. Furthermore, LL-37 is suggested to bind with CD14 near the LPS binding site and inhibit the binding of LPS to CD14 molecule [20], [22], [42]. In this study, LL-37 strongly inhibited the binding of LPS to J774 cells (Fig. 4). Moreover, we confirmed that the LPS binding to J774 cells is mostly mediated by the binding with CD14/TLR4, based on the findings that the LPS binding was substantially suppressed by both 4C1 (a CD14 blocking antibody) and MTS510 (a TLR4/MD-2 complex blocking antibody) [20]. Together these observations suggest that LL-37 suppresses the LPS/ATP-induced pyroptosis by inhibiting the binding of LPS to its receptor CD14/TLR4.

Furthermore, we confirmed that ATP stimulates the nucleotide receptor P2X_7_ to activate caspase-1 in J774 cells and the activation was inhibited by the P2X_7_ antagonists KN-62 and KN-93 (Fig. 5). In this study, LL-37 (1 µg/ml = 0.22 µM) significantly suppressed the ATP-induced caspase-1 activation, suggesting that LL-37 inhibits the ATP-induced P2X_7_ activation. Consistent with our observation, LL-37 (1∼10 µM) is reported to inhibit the P2X_7_-mediated uptake of extracellular calcium by murine salivary gland cells in response to ATP [33]. In contrast, cathelicidins are reported to act as an agonist for P2X_7_; i.e., LL-37 and CRAMP (10∼20 µM) activate P2X_7_ to induce the IL-1β production, caspase-1 activation and inflammasome formation in LPS-primed macrophages [40], [43], [44]; LL-37 (0.01∼5 µg/ml) activates P2X_7_ to suppress the apoptosis of human neutrophils [45]. However, we could not detect the agonistic effect of LL-37 on the P2X_7_-mediated caspase-1 activation in J774 cells under our experimental conditions (LL-37, 0.01∼1 µg/ml; data not shown). Moreover, we could not determine the effect of higher concentrations of LL-37 on P2X_7_ activation, because LL-37 was obviously cytotoxic to J774 cells at >5 µg/ml (data not shown). Importantly, LL-37, an amphipathic peptides, has been reported to form an α-helix in aqueous buffer and potently bind to P2X_7_ with a 2.99 kcal/mol binding potential [34], [44]. Moreover, oxidized ATP, an irreversible P2X_7_ blocker, which interacts with unprotonated lysine residues located in the vicinity of the ATP binding site of the receptor, inhibits the action of LL-37 on P2X_7_ [45], [46]. Based on these observations, it is tempting to speculate that LL-37 interacts with the ligand-binding site of P2X_7_ and suppresses the subsequent binding and action of ATP, resulting in the partial inhibition of LPS/ATP-induced macrophage pyroptosis.

Interestingly, LPS stimulation alone was enough for the expression of IL-1β mRNA, and LL-37 significantly inhibited the IL-1β mRNA expression as well as the binding of LPS to its receptor (CD14/TLR4) at >0.1 µg/ml. In contrast, caspase-1 activation, inflammasome formation and cell death were not essentially induced by LPS stimulation alone but considerably induced by the subsequent stimulation of LPS-primed cells with ATP, and LL-37 significantly suppressed these pyroptotic features (caspase-1 activation, inflammasome formation and cell death) as well as the ATP-induced P2X_7_ activation at 1 µg/ml but not 0.1 µg/ml. These observations likely suggest that LL-37 can strongly suppress the CD14/TLR4-mediated binding of LPS (Fig. 4) and signaling (Fig. 2B), due to its potent LPS neutralizing activity based on the high-binding affinity for LPS [47]; however, LL-37 can only partially inhibit the P2X_7_-mediated signaling by ATP during the process of LPS/ATP-induced pyroptosis.

During pyroptosis, macrophages and dendritic cells rapidly produce and extracellularly release IL-1β accompanied with their cell death. IL-1β is a prototypical proinflammatory cytokines, which stimulates both local and systemic inflammatory/immune responses [12]. Thus, the blocking of IL-1 with IL-1 receptor antagonist is reported to protect animals from lethal endotoxemia and Gram-negative sepsis [9] and patients from proven bacteremia [48]. Moreover, dying macrophages release alarming factors into the environment and elicit the dysregulated inflammation/immune responses; for example, ATP is extracellularly released from dying cells and induces the production of nitric oxide and reactive oxygen species, formation of inflammasome and activation of caspase-1 in macrophages [23], [49], [50]. Caspase-1 is responsible for the IL-1β release and cell death (pyroptosis) [11]. Importantly, the knock-down of caspase-1 gene reduced splenic cell death in an *E. coli* induced-sepsis model [14] and HMGB-1 release in an LPS induced-endotoxemia model [16], [51], and improved the survival rates in both models. Thus, caspase-1 could be an effective target for the treatment of sepsis. Furthermore, LPS acts as a potent inducer of proinflammatory cytokine production by inflammatory/immune cells, and is recognized as a key molecule in the pathogenesis of Gram-negative sepsis [20]. Consequently, the clearance or neutralization of LPS could also be a target for the sepsis treatment.

Moreover, P2X_7_ plays an important role in various inflammatory diseases by producing proinflammatory cytokines (IL-1β and IL-18) via the activation of caspase-1 [52], [53], [54], and now regarded as a novel target for the sepsis treatment, since the blockage of P2X_7_ by an antagonist increases the survival of *E. coli* induced-septic models [55]. In this context, the present study indicated that the stimulation with LPS and ATP induces the pyroptosis (cell death accompanied with caspase-1 activation and IL-1β release) of macrophage-like J774 cells, and LL-37 inhibits the pyroptosis by dual mechanism involving the neutralization of LPS and the inhibition of P2X_7_ responses to ATP. Moreover, we confirmed that LL-37 suppresses the LPS/ATP-induced release of IL-1β and LDH from human macrophages (PMA-primed THP-1 cells) (Fig. S1A and B). We also performed a preliminary experiment to evaluate the potential effect of LL-37 on sepsis using a murine cecal ligation and puncture (CLP) [56] by the intravenous administration of LL-37 (2 µg/mouse) into mice after CLP. Importantly, the mortality was 100% for the CLP group without LL-37 administration (n = 5), but was reduced to 60% for the LL-37-administered group (n = 5) at 48 h after CLP. Moreover, LL-37 administration reduced the IL-1β level in the peritoneal fluid (CLP group, 986±340 pg/ml, n = 3 *vs.* LL-37-treated group, 354±195 pg/ml, n = 3), and suppressed the pyroptosis of F4/80 positive peritoneal macrophages (CLP group, 4.8±1.3 %, n = 3 *vs.* LL-37-treated group, 2.1±0.3 %, n = 3) at 15 h after the CLP, compared with the CLP group. Thus, LL-37 is expected to suppress the pyroptosis of macrophages, thereby possibly modulating the dysregulated inflammation/immune responses and exerting a protective action in sepsis.

LL-37 is originally identified as an AMP, which participates in the innate immune system, with potentials to protect host from invasive microbial infections and neutralize Gram-negative LPS [20], [29], [39], and now regarded as a multifunctional molecule that links the innate immune response to the adaptive immune system by exerting diverse immunomodulatory actions, such as production of cytokines and chemokines, induction of immune cell chemotaxis, and alteration of dendritic cell differentiation [26], [30], [57], [58]. The present study has demonstrated for the first time an additional function of LL-37 to suppress the pyroptosis of macrophage-like cells by neutralizing LPS action and inhibiting P2X_7_ response. Furthermore, this finding may provide a new direction for the drug design of immunomodulatory peptides utilizing LL-37 as a lead compound with a dual action on the LPS binding and P2X_7_ activation.

## Supporting Information

Table S1 **Gene specific PCR primers and number of PCR cycles.**
(TIF)

Figure S1 **Effect of Ac-YVAD-CHO on the TNF-α production by LPS/ATP-treated J774 cells.** J774 cells were primed with 10 ng/ml LPS for 4 h, and then treated with 3 mM ATP for 90 min in the absence or presence of 20 µM Ac-YVAD-CHO (a caspase-1 inhibitor). Cells were also incubated with LPS alone, or without LPS, ATP and Ac-YVAD-CHO. Thereafter, the supernatants were recovered for the assay of TNF-α. TNF-α 1evels were determined using a commercially available mouse TNF-α ELISA kit. Data shows the mean ± SD of 3 separate experiments. Values are compared between the absence and presence of LPS treatment.
(TIF)

Figure S2 **Effect of LL-37 on the LPS/ATP-induced pyroptosis of THP-1 cells.** PMA-differentiated THP-1 cells were primed with 1 µg/ml LPS for 24 h in the absence or presence of LL-37 (1 µg/ml), and treated with 5 mM ATP for 4 h. Cells were also incubated with LPS or ATP alone, or without LPS, ATP and LL-37. Thereafter, the supernatants were recovered for the assays of IL-1β (A) and LDH (B). Data shows the mean ± SD of 3 separate experiments. Values are compared between the absence and presence of LL-37. *P<0.05, ***P<0.001.
(TIF)
