# Supplementary figures and images for: Antimicrobial Cathelicidin Peptide LL-37 Inhibits the LPS/ATP-Induced Pyroptosis of Macrophages by Dual Mechanism
Source: PLoS One. 2014 Jan 16;9(1):e85765. doi: 10.1371/journal.pone.0085765 (PMC3894207; doi:10.1371/journal.pone.0085765)

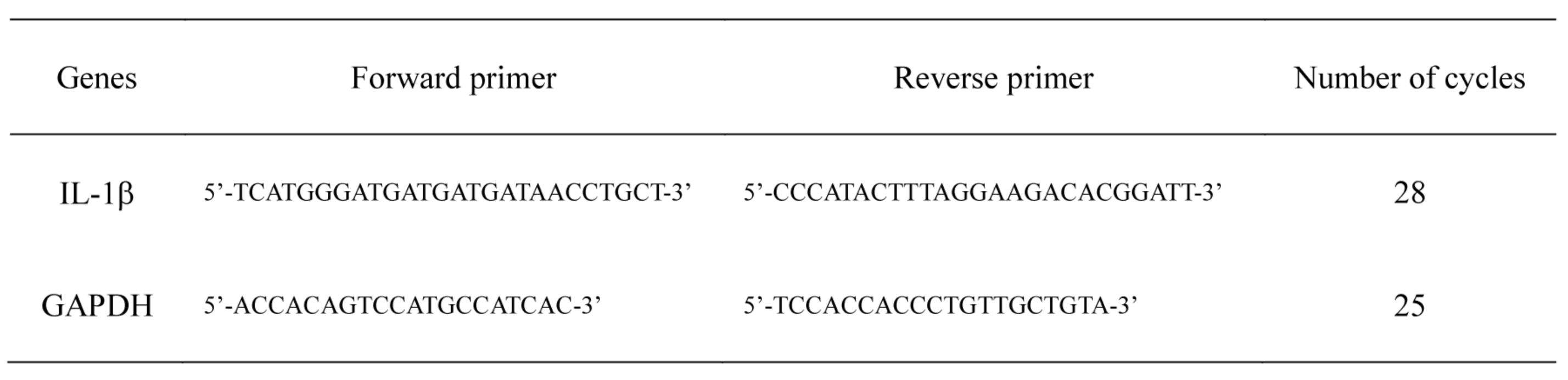

Supplement: Table S1 — Gene specific PCR primers and number of PCR cycles. (TIF) [file pone.0085765.s001.tif]

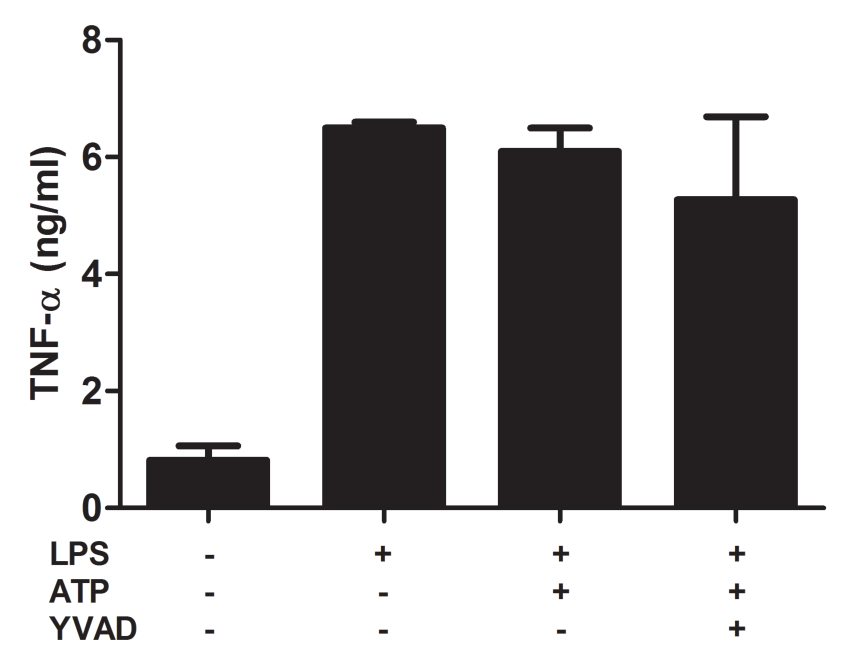

Supplement: Figure S1 — Effect of Ac-YVAD-CHO on the TNF-α production by LPS/ATP-treated J774 cells. J774 cells were primed with 10 ng/ml LPS for 4 h, and then treated with 3 mM ATP for 90 min in the absence or presence of 20 µM Ac-YVAD-CHO (a caspase-1 inhibitor). Cells were also incubated with LPS alone, or without LPS, ATP and Ac-YVAD-CHO. Thereafter, the supernatants were recovered for the assay of TNF-α. TNF-α 1evels were determined using a commercially available mouse TNF-α ELISA kit. Data shows the mean ± SD of 3 separate experiments. Values are compared between the absence and presence of LPS treatment. (TIF) [file pone.0085765.s002.tif]

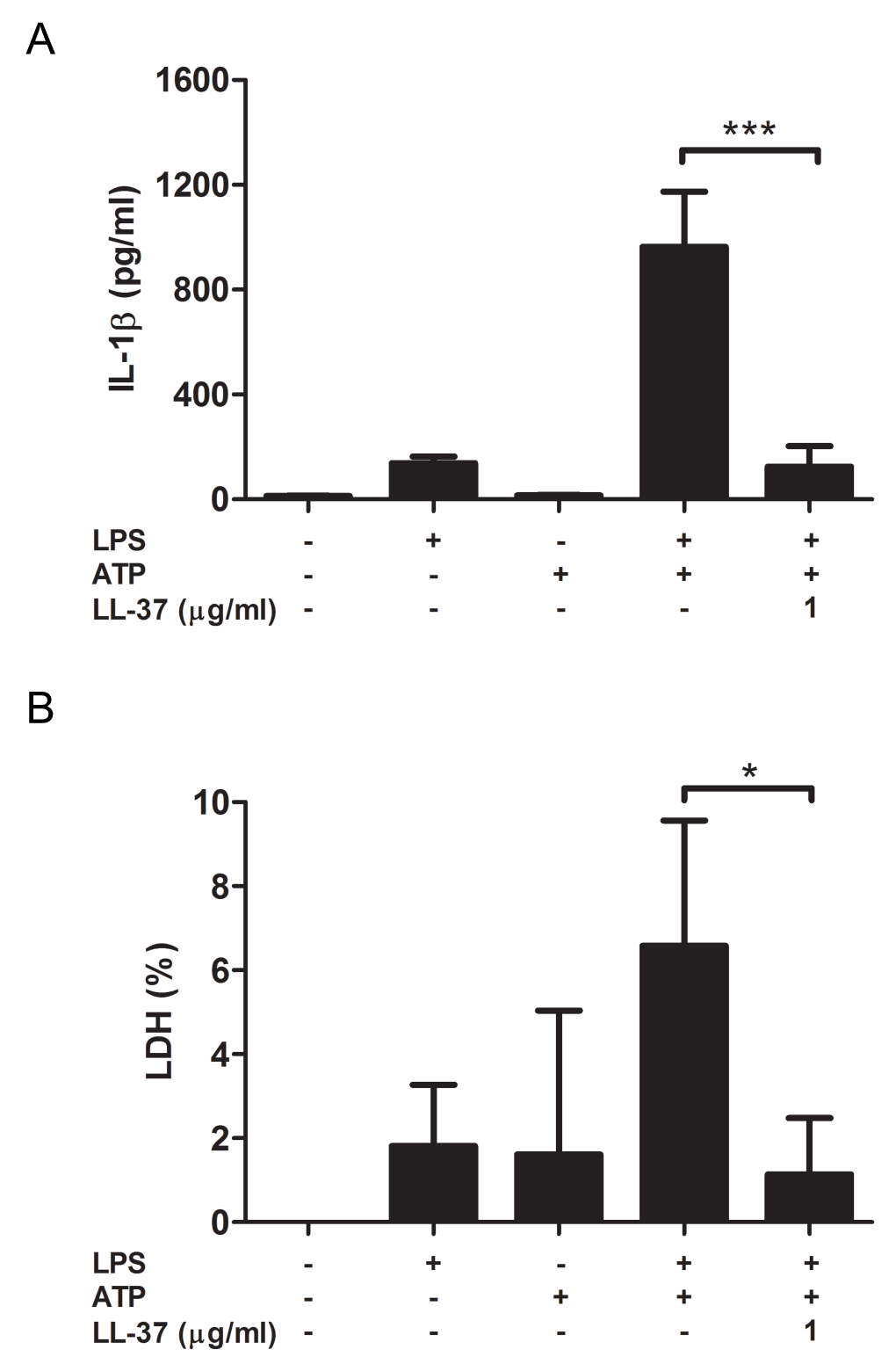

Supplement: Figure S2 — Effect of LL-37 on the LPS/ATP-induced pyroptosis of THP-1 cells. PMA-differentiated THP-1 cells were primed with 1 µg/ml LPS for 24 h in the absence or presence of LL-37 (1 µg/ml), and treated with 5 mM ATP for 4 h. Cells were also incubated with LPS or ATP alone, or without LPS, ATP and LL-37. Thereafter, the supernatants were recovered for the assays of IL-1β (A) and LDH (B). Data shows the mean ± SD of 3 separate experiments. Values are compared between the absence and presence of LL-37. *P<0.05, ***P<0.001. (TIF) [file pone.0085765.s003.tif]
